# Physical activity, sedentary behavior time and lipid levels in the Observation of Cardiovascular Risk Factors in Luxembourg study

**DOI:** 10.1186/s12944-015-0085-3

**Published:** 2015-08-11

**Authors:** Georgina E Crichton, Ala’a Alkerwi

**Affiliations:** Nutritional Physiology Research Centre, University of South Australia, GPO Box 2471, Adelaide, 5001 Australia; Luxembourg Institute of Health, Grand-Duchy of Luxembourg, Luxembourg, Luxembourg

**Keywords:** Sedentary behavior time, Physical activity, HDL-cholesterol, LDL-cholesterol, Triglycerides

## Abstract

**Background:**

Recently attention has been drawn to the health impacts of time spent engaging in sedentary behaviors. While many studies have investigated general physical activity (PA) in relation to blood lipid levels, the current study aimed to examine the intensity of activity, including sedentary behavior time, and time spent engaging in moderate and intense PA, with concentrations of HDL and LDL-cholesterol, total cholesterol, and triglycerides.

**Methods:**

Participants comprised 1331 individuals, aged 18 to 70 years, from the Observation of Cardiovascular Risk Factors in Luxembourg (ORISCAV-LUX) study, who underwent objective cardiovascular health assessments and completed the International Physical Activity Questionnaire (IPAQ). Time spent engaging in sedentary behaviors (*screen time* on a workday and a day off, and total sitting time on a work day), and moderate and intense PA, were related to levels of HDL and LDL-cholesterol, total cholesterol, and triglycerides. Analyses were conducted in the whole sample, and then with stratification according to BMI (normal weight versus overweight/obese).

**Results:**

Both lower *screen time* during days off and higher *intense PA* time were significantly associated with higher HDL-cholesterol after full adjustment for socio-demographic factors, dietary factors and smoking (both p < 0.05). In normal weight individuals, consistent positive relations between triglycerides, LDL, and total cholesterol with all sedentary behavior time variables were observed (all p < 0.05; adjusted for age, education, gender). There were no statistically significant associations between any intensity level of PA or sedentary behavior time variable and lipid levels in those overweight or obese.

**Conclusions:**

Spending less time in sedentary behaviors, and engaging in medium levels of intense physical activity may be associated with a more favorable blood lipid profile, particularly with regard to levels of HDL and triglycerides.

## Background

Physical activity (PA) has been inversely related to fatal and non-fatal cardiovascular disease (CVD) [1–6]. Attention has more recently been drawn to the association between sedentary behaviors and negative health outcomes, including CVD [7–15]. Sedentary behavior refers to any waking behavior that involves an energy expenditure of less than 1.5 metabolic equivalent units (METs) [16, 17]. This can include activities undertaken in sitting or lying, such as watching television or using a computer. It has been previously demonstrated that greater times spent sitting, viewing television and using a computer may be unfavorably associated with ideal cardiovascular health [18]. However, there are some inconsistencies in the literature [19, 20] and several reviews have concluded that causal relationships between sedentary behavior time and health outcomes need to be further clarified [21, 22].

Low-density lipoprotein cholesterol (LDL) is an atherogenic, harmful lipoprotein, responsible for the atherosclerotic process and an increased risk for CVD [23, 24]. Elevated blood triglycerides have a dose-dependent association with cardiovascular-related and all-cause mortality [25]. In contrast, high-density lipoprotein cholesterol (HDL) is related to lower prevalence of cardiovascular mortality [26] and incidence of coronary heart disease (CHD) [27, 28]. In contrast to the research focused on relations between lipid levels and PA, fewer studies to date have examined associations between sedentary behavior time and lipids.

The ATTICA study showed that physically active women had significantly lower levels of total cholesterol, LDL-cholesterol, triglycerides, and higher levels of HDL- cholesterol, compared to sedentary women [29]. Significance was only retained for HDL associations after adjustment for age, smoking, and body mass index (BMI). Clinical trials have shown that physical exercise may increase HDL-cholesterol in healthy elderly subjects [30] and further may facilitate increases in HDL-cholesterol from dieting in overwight individuals [31]; while others suggest that diet and exercise interventions in overweight and obese adults may have differential effects on LDL- versus HDL-cholesterol [32]. One recent study by Aadland et al. [33] examined associations between lipoprotein concentrations and PA versus sedentary behavior in healthy adults. Their results suggested differential effects of sedentary versus PA time, as they found positive associations between sedentary time and LDL, total cholesterol and triglycerides, and between moderate-to-vigorous physical activity (MVPA) and HDL-cholesterol. However, this study had a small sample size of 73 subjects and did not control for other potential confounding factors. Other studies have shown conflicting results; sedentary behavior time has not been associated with any lipoprotein cholesterol or triglycerides [34, 35], associated with higher triglycerides, total and LDL-cholesterol and lower HDL-cholesterol [36], and with both higher HDL-cholesterol and triglycerides [7]. None of these studies that we are aware of have specifically differentiated whether any associations between sedentary behavior time and lipid levels vary according to day of the week (i.e., a workday versus a day off), or have stratified according to BMI.

Further, a recent meta-analysis has shown a positive association between sedentary behaviour time and odds of having metabolic syndrome by 73 % [37], of which raised triglycerides and reduced HDL-cholesterol are two risk factors [38]. This meta-analysis was based on 10 cross-sectional studies and indicated a significant influence of sedentary time on disease risk, independent of physical activity [37].

The first aim of this study was to investigate sedentary time (television and computer time) both on a weekday and a day off, in relation to total-, HDL- and LDL-cholesterol, and triglycerides. The second aim was to explore relations between moderate and intense PA time and lipid levels. It was hypothesised that there would be positive associations between sedentary behaviors and lipid levels, and inverse associations between PA and lipids. A hypothesis as to how any relationships may vary according to day of the week or according to body weight status was not advanced.

## Methods

### Participants

Data was obtained from the Observation of Cardiovascular Risk Factors in Luxembourg (ORISCAV-LUX), a nationwide cross-sectional study conducted, to gather information on the prevalence of cardiovascular risk factors, and lifestyle health-related factors such as PA and nutritional status, among the general adult population of Luxembourg. A representative random sample of 1432 individuals, stratified by sex, age (18–69 years) and district of residence, selected from the national health insurance registry was recruited between November 2007 and January 2009. This sample size was estimated to ensure statistical power, i.e. a statistical precision of at least 2 % for the estimation of the prevalence of the risk factors at the 95 % confidence level. Institutionalized subjects (n = 12), pregnant women (n = 21), subjects with serious mental and/or physical handicap (n = 5), prisoners (n = 1), people outside the determined age range (n = 2) and those deceased before recruitment (n = 5) were excluded. Description of the study design and recruitment have been published in detail previously [39, 40]. A total of 1432 participants completed the recruitment procedure. After eliminating those with missing data on lipids, sedentary behavior time, PA or covariates, data from 1331 participants were available for the present analysis.

### Procedure and measures

#### Demographics and health information

Information on demographic and socioeconomic characteristics, including age, gender, education, occupation, and income was obtained from a detailed self-administered questionnaire. Education level was classified into three levels, based on the highest diploma obtained: ‘primary’ (less than 12 years of education), ‘secondary’ (approximately 12 to 13 years of education) and ‘tertiary’ (more than 13 years). Participants were required to indicate their occupation, and based on the type of work performed, each participant was categorized into one of three groups with regard to their occupation: ‘sedentary’, ‘moderately active’, and ‘active’. For example, manual laborers were placed in the active category, while scientific professionals were placed in the sedentary category. Economic status was ascertained by asking participants to select the category best representing total monthly household income. Body weight was measured using a digital column scale (Seca® 701, Hamburg, Germany), recorded to the nearest 0.1 kg, with subject barefoot and wearing light clothing. Standing body height was recorded to the nearest 0.2 cm with a portable wall stadiometer (Seca, Germany). BMI was calculated as weight in kg divided by height in metres squared (kg/m^2^).

#### Lipid levels

Blood samples were collected after a minimum 8-hour fast from the antecubital vein to measure total cholesterol (mg/dL), HDL-cholesterol (HDL, mg/dL) and LDL-cholesterol (LDL, mg/dL), and triglycerides (mg/dL), by using Roche (Switzerland) reagents on a P module of a Modular analyzer (Roche, Switzerland) [41]. All biochemical analyses were carried out within two hours of blood sampling in the core laboratory of ‘Centre Hospitalier du Luxembourg (CHL)’. The CHL laboratory applies strict internal and external standard quality control techniques.

#### Physical activity and sedentary behavior time

PA and sedentary behaviour time was assessed using the short-form International Physical Activity Questionnaire (IPAQ) [42], designed and validated to measure PA in large populations. The IPAQ is conceived in a way to distinguish the time spent in performing four types of physical behaviors (vigrous physical activity, moderate physical activity, walking and sitting).

#### Physical activity

Participants were required to report the number of days in the preceding week (including the weekend) that they engaged in both moderate and intense PA, and the amount of time spent in these activities on one of these days (in hours). Examples of moderate activities provided included carrying light loads, vacuuming or slow cycling, whilst intense activities included carrying heavy loads, digging or playing soccer.

Mean daily moderate and intense PA time (in hours per day) was calculated by multiplying self-reported time spent engaging in each with the reported number of days per week in which these activities were undertaken, and dividing by seven. These values were summed to obtain total mean PA time (moderate plus intense). Moderate, intense and total PA time were then categorized into three groups: <0.5 hours per day, 0.5-1 hour per day, and >1 hour per day.

#### Sedentary behavior time

Sedentary behaviour time was also obtained from the IPAQ. Participants were required to report *screen time*, i.e., how much time they spent watching television (including videos/DVD), and in front of a computer (including internet and video games), during a typical workday, and during a day off. Participants also reported *overall sitting time*, i.e., how much time they spent sitting during a normal weekday (distinguished form a weekend day), including time spent sitting at place of work, on transportation, reading, visiting friends’, sitting or laying down to watch television or use a computer. All responses were given in hours per day, with reference to the preceding seven days.

Total *screen time* for a workday and a day off were calculated by summing television time and computer time. The *screen time* variables were then divided into three categories: ≤1, >1-4, ≥4 hours per day. The *overall sitting time* variable was divided into five categories: 0–2, >2-4, >4-6, >6-10, and >10 hours per day.

In addition, individuals were categorized into one of four groups to reflect their mean weekly behavior with regard to activity level, referred to as *physical activity status*. Participants who reported moderate or intense PA on at least one day per week were classified into low, medium or high tertiles of PA, calculated from the total PA time variable (hours per day). The remainding participants who did not engage in moderate or intense PA during the preceding week were classified as ‘sedentary’. This evaluation method of overall PA has been used previously [29].

#### Other covariates

Detailed data regarding cigarette smoking were obtained from the questionnaire. Each participant was classified as current smoker, ex-smoker or non-smoker. A validated, semi-quantified food frequency quesionnaire (FFQ) was used to assess the frequency of consumption of 134 food and beverage items over the previous three months [43, 44]. Participants were asked how frequently they consumed one standardized portion of each food, with six frequency response categories ranging from “never or rarely” to “2 or more times per day”. Energy and nutrient intake data were compiled to obtain total carbohydrate (g/day), total protein (g/day), total fat (g/day), total fiber (g/d), alcohol (g/day), and total energy intake (Kcal/day). Total carbohydrate, protein, total and saturated fat as proportions of total enery intake were subsequently calculated and expressed as % E.

### Statistical analyses

For the descriptive analyses, Chi-squared tests and ANOVA were performed to compare the socio-demographic characteristics, blood lipid levels and dietary characteristics of participants according to the overall activity level classification, ranging from sedentary to a high level of PA.

General linear modelling with polynomial trend analyses was used to compare each lipid biomarker (as continuous variables) across increasing categories of *screen time* (workday and day off), *total sitting time*, and PA time (*moderate* and *intense*). Two covariate sets were employed:

1. Basic: age, education, gender.
2. Extended: basic model in addition to income, profession, carbohydrate % E, protein % E, saturated fat % E, alcohol (ml/day), and smoking.

Total PA (hours/day) was included in the extended model for analyses with the sedentary behavior time variables only.

The same statistical procedures, assessing relations between sedentary behavior time, PA time and blood lipids, were performed separately in the sample stratified by BMI status: normal weight (BMI < 25 kg/m^2^, n = 589), and overweight/obese (BMI ≥ 25 kg/m^2^, n = 741).

All statistical anlyses were performed with PASW for Windows® version 21.0 software (formerly SPSS Statistics Inc. Chicago, Illinois). P < 0.05 was considered statistically significant.

### Ethical statement

All participants gave informed written consent to take part in the study. The study design and information collected were approved by the National Research Ethics Committee (Comité d'Ethique de Recherche, CNER) and the National Commission for Private Data Protection (Commission Nationale pour la Protection des Données, CNPD).

## Results

### Sample characteristics

The sample consisted of 1331 individuals (646 males and 685 females), aged 18 to 69 years (mean 44.4 years). Mean *overall sitting time* was 6.2 (±3.2) hours. Reported *screen time* (television plus computer time) on a weekday was 4.2 (±3.4) hours, and 3.4 (±2.5) hours on a day off. Mean daily *moderate PA* time was 1.4 (±2.0) hours, and 0.4 (±0.9) hours for *intense PA*.

Overall, 11 % of participants were classified as sedentary, with a higher proportion of men (66.7 %) than women (33.3 %) being inactive (p < 0.001). Sedentary participants were older than those who were physically active (p < 0.05), but frequency of active smoking was similar between sedentary participants (25.3 %) and active participants (Table 1).

**Table 1:** Demographic and socioeconomic characteristics according to physical activity status^a^, in ORISCAV-LUX participants (*N* = 1331)

	Physical activity status^a^				
Characteristic	Sedentary n = 150	Low (>0-0.5 hours/day) n = 393	Medium (>0.5-2 hours/day) n = 394	High (>2 hours/day) n = 394	p^b^
	%	%	%	%	
Gender					<0.001
Males	66.7	47.6	43.9	47.2	
Females	33.3	52.4	56.1	52.8	
Education					<0.001
No diploma	35.4	20.6	22.8	31.2	
Secondary	39.5	42.0	47.7	54.7	
Tertiary	25.2	37.4	29.4	14.1	
Occupation					<0.001
Sedentary	4.0	6.1	5.1	3.3	
Moderately active	73.3	78.1	78.4	66.8	
Active	21.3	15.3	16.2	29.2	
Income					<0.001
Q1	26.4	20.7	14.1	21.7	
Q2	44.8	41.6	53.2	58.3	
Q3	23.2	29.3	27.9	17.7	
Q4	5.6	8.4	4.9	2.3	
Smoking, current	25.3	17.6	17.8	26.4	0.003

^a^Sedentary time: reported undertaking no moderate or intense physical activity on any day of the preceding week; low, medium and high groups: tertiles according to total moderate plus intense physical activity (hours per day)

^b^Chi-square for categorical variables

Compared to sedentary participants, physically active participants had lower levels of total cholesterol, LDL-cholesterol, and triglycerides (all p < 0.05, linear trend), and significantly higher HDL-cholesterol (p < 0.05) (Table 2). The three physically active groups also had lower mean BMI, waist circumference, systolic and diastolic BP, than the sedentary group (all p < 0.05).

**Table 2:** Health and dietary characteristics according to weekday sitting time^a^, in ORISCAV-LUX participants (*N* = 1331)

	Physical activity status^a^									
Characterstic	Sedentary n = 150		Low (>0-0.5 hours/day) n = 393		Medium (>0.5-2 hours/day) n = 394		High (>2 hours/day) n = 394		Partial eta squared	p (linear)^b^
	*M*	*SD*	*M*	*SD*	*M*	*SD*	*M*	*SD*		
Age (years)	47.2	12.9	43.5	13.5	43.9	12.8	44.7	12.9	0.007	0.064
BMI (kg/m^2^)	28.2	6.1	26.3	5.0	26.0	4.7	26.6	4.7	0.017	0.001
Waist circmference (cm)	95.6	15.9	89.1	14.0	87.9	12.9	89.2	13.1	0.026	<0.001
Systolic BP (mm Hg)	134.4	18.8	128.5	17.8	128.4	16.7	130.6	18.3	0.011	0.028
Diastolic BP (mm Hg)	85.7	11.2	81.8	11.0	81.6	10.6	82.5	11.6	0.013	0.003
Lipid biomarkers										
Total cholesterol (mg/dL)	208.8	44.1	201.1	40.7	201.9	42.2	199.4	37.7	0.004	0.023
HDL cholesterol (mg/dL)	57.1	16.8	62.7	18.4	63.5	16.9	60.2	15.1	0.015	0.047
LDL cholesterol (mg/dL)	131.9	39.1	122.1	35.4	123.4	35.3	124.1	32.9	0.007	0.034
Triglycerides (mg/dL)	134.4	86.6	120.8	115.7	109.5	92.6	109.3	73.2	0.008	0.002
Dietary factors										
Total energy intake (kCal)	2366	973	2311	865	2443	960	2530	948	0.009	0.024
Carbohydrate, (% E)	42.4	7.6	42.1	7.4	42.0	7.3	43.4	7.6	0.006	0.22
Protein (% E)	16.3	3.6	16.0	3.2	16.0	3.1	15.8	3.2	0.002	0.11
Total fat (% E)	37.2	7.2	38.9	6.9	39.1	6.8	37.9	7.4	0.008	0.29
Saturated fat (% E)	12.9	3.0	13.6	2.8	13.6	2.8	13.0	2.9	0.013	0.83
Alcohol (ml/day)	11.4	13.4	9.1	12.2	8.0	10.1	7.8	10.7	0.010	0.001

^a^Sedentary time: reported undertaking no moderate or intense physical activity on any day of the preceding week; low, medium and high groups: tertiles according to total moderate plus intense physical activity (hours per day)

^b^ANOVA for continuous variables

### Sedentary behavior time and lipids

In the whole sample, there was a significant, inverse association observed between *screen time* during a day off and HDL-cholesterol (Table 3). Those who engaged in at least four hours of *screen time* per day (television viewing and using a computer) had significantly lower HDL-cholesterol levels (mean 60.4 ± 0.75 mg/dL/ [1.56 ± 0.02 mmol/L]) than those who spent one hour or less undertaking these behaviors (mean 63.6 ± 1.2 mg/dL [1.65 ± 0.03 mmol/L]) (p < 0.05). This association was significant after taking into account sociodemographic factors (age, education, gender, income, profession), dietary intake, total PA (moderate plus intense PA, hours/day) and smoking.

**Table 3:** Multivariate adjusted mean and SE of HDL, LDL, and total cholesterol, and triglycerides across increasing sedentary and physical activity time categories in ORISCAV-LUX (*N* = 1331)

Lipid outcome^a^	Model	Screen time (day off)						*R* ^*2*^	*p* linear
		≤1 hour/day 13.9 %		>1-4 hours/day 48.7 %		≥4 hours/day 37.3 %			
		*M*	*SE*	*M*	*SE*	*M*	*SE*		
HDL-cholesterol	Basic	63.8	1.1	61.7	0.59	60.4^*^	0.69	0.22	0.011
	Extended^b^	63.6	1.2	61.6	0.62	60.4^*^	0.75	0.24	0.030
LDL-cholesterol	Basic	121.3	2.5	123.6	1.31	126.2	1.5	0.12	0.09
	Extended^b^	122.3	2.8	124.3	1.4	127.3	1.7	0.14	0.13
Total cholesterol	Basic	199.5	2.9	200.6	1.5	203.8	1.8	0.10	0.21
	Extended^b^	201.0	3.3	201.7	1.7	204.7	2.0	0.11	0.34
Triglycerides	Basic	102.8	6.5	112.9	3.4	121.4^*^	3.9	0.06	0.016
	Extended^b^	104.8	7.3	114.5	3.7	120.6	4.5	0.09	0.07
		PA time (intense)							
		<0.5 hours/day 73.5 %		0.5-1 hours/day 13.7 %		>1 hour/day 12.5 %			
		*M*	*SE*	*M*	*SE*	*M*	*SE*		
HDL-cholesterol	Basic	60.9	0.48	64.0^*^	1.1	62.5	1.2	0.21	0.031^a^
	Extended^c^	60.8	0.52	64.5^*^	1.2	61.8	1.2	0.24	0.016^a^
LDL-cholesterol	Basic	124.5	1.1	124.6	2.4	122.0	2.6	0.11	0.37
	Extended^c^	125.4	1.2	125.7	2.7	122.4	2.8	0.12	0.32
Total cholesterol	Basic	202.4	1.2	202.5	2.9	197.3	3.0	0.09	0.12
	Extended^c^	203.4	1.4	204.1	3.2	197.6	3.3	0.10	0.10
Triglycerides	Basic	120.4	2.9	105.7^*^	6.8	97.6^*^	7.2	0.06	0.003
	Extended^c^	121.3	3.3	108.4	7.6	97.8^*^	7.9	0.08	0.006

^*^significantly different from ≤ 1 hour/day group (for screen time) or <0.5 hours/day group (for PA), *p* < 0.05

^a^overall *p* value

Basic: age, education, gender

^b^Extended: Basic + income, profession, total PA (hours/day), carbohydrate as % of total enery intake, protein as % of total energy intake, saturated fat as % of total energy intake, alcohol, smoking

^c^Extended: Basic + income, profession, carbohydrate as % of total enery intake, protein as % of total energy intake, saturated fat as % of total energy intake, alcohol, smoking

There was a significant, positive association between triglyceride levels and *screen time* during a day off (p < 0.05 basic model, linear trend, Table 3). Those in the highest *screen time* category had significantly higher triglyceride levels (121.4 ± 3.9 mg/dL [1.37 ± 0.04 mmol/L]) than those in the lowest category (102.8 ± 6.5 mg/dL [1.16 ± 0.07]) (p < 0.05), after adjustment for age, sex and education level. Although the same linear increase in triglyceride levels was observed with the addition of further confounding variables (extended model), the association was no longer statistically significant (p = 0.07).

### Moderate and intense physical activity time and lipid levels

Those who undertook a medium level of *intense PA* (0.5-1 hour/day) had significantly higher HDL-cholesterol levels (mean = 64.5 ± 1.2 mg/dL [1.67 ± 0.03 mmol/L]) than those who undertook less than this (mean = 60.8 ± 0.52 mg/dL [1.57 ± 0.01 mmol/L]) (p < 0.05, extended model, Table 3). Triglyceride levels decreased in a linear pattern with increasing duration of *intense PA* (p < 0.01, extended model). Both of these associations remained significant after adjustment for sociodemographics, dietary factors, and smoking. There were no significant associations found between total or LDL-cholesterol and *intense PA* time. Non of the lipid biomarkers were significantly associated with *moderate PA* time (data not shown).

### Stratification according to weight status

#### BMI < 25 kg/m^2^

In normal weight participants (BMI < 25 kg/m^2^, n = 589), significant, linear associations were observed between increasing *screen time* (day off) and total cholesterol, LDL-cholesterol, and triglycerides (all p < 0.05, basic model, Table 4). Those who reported the least *screen time* (≤1 hour/day) had significantly lower levels of all three lipids, compared to the highest *screen time* group (≥4 hours/day) (all p < 0.05, basic models). Associations remained for LDL-cholesterol and triglycerides with full adjustment, but significance was lost for total cholesterol.

**Table 4:** Multivariate adjusted mean and SE of HDL-, LDL-, and total cholesterol, and triglycerides across increasing sedentary and PA time categories in ORISCAV-LUX, in normal weight participants (BMI <25 kg/m^2^, *n* = 589)

Lipid outcome^a^	Model^b^	Screen time (day off)						*R* ^*2*^	*p* linear
		≤1 hour/day 13.9 %		>1-4 hours/day 48.7 %		≥4 hours/day 37.3 %			
		*M*	*SE*	*M*	*SE*	*M*	*SE*		
HDL-cholesterol	Basic	70.0	1.7	67.8	0.9	66.3	1.1	0.19	0.07
	Extended	70.2	1.8	68.1	0.9	66.7	1.3	0.23	0.12
LDL-cholesterol	Basic	109.1^*^	3.2	112.9	1.7	118.4	2.2	0.20	0.020
	Extended	110.0^*^	3.5	114.0	1.9	119.3	2.5	0.23	0.036
Total cholesterol	Basic	188.6^*^	3.8	190.8^*^	2.0	198.1	2.5	0.22	0.039
	Extended	190.2	4.0	192.3	2.1	198.6	2.9	0.26	0.10
Triglycerides	Basic	76.7^*^	4.8	81.0^*^	2.5	97.9	3.2	0.07	<0.001
	Extended	77.3^*^	5.0	82.3^*^	2.7	96.7	3.5	0.11	0.002

^*^significantly different from ≥ 4 hours/day group, *p* < 0.05

Basic: age, education, gender

Extended: Basic + income, profession, total PA (mins/wk), carbohydrate as % of total enery intake, protein as % of total energy intake, saturated fat as % of total energy intake, alcohol, smoking

A similar pattern of results were observed for total cholesterol and triglycerides with the work day sedentary behavior time variables. Both total cholesterol and triglycerides increased with increasing *screen time* on a work day and *overall sitting time* (all p < 0.05, linear trends, basic models only, data not shown).

Neither *moderate* or *intense PA* time was related with any lipid in normal weight participants (data not shown).

#### BMI ≥ 25 kg/m^2^

There were no observed associations between any of the sedentary behavior time variables and lipid biomarkers in overweight or obese participants. Neither *moderate* or *intense PA* time was related with any lipid in the overweight/obese groups.

## Discussion

In this cross-sectional population-based study of European adults, *screen time* (viewing television and using a computer) on a day off was inversely associated with HDL-cholesterol, regardless of age, gender, education, profession type, income, PA, dietary factors, and smoking. *Intense PA* time was positively associated with HDL-cholesterol, and inversely associated with triglyceride levels (both with full adjustment). Relations between the other lipids and activity level were only observed in normal weight (BMI < 25 kg/m^2^) individuals. For these participants, consistent positive associations with *screen time* on both a work day and a day off, and total sitting time on a work day with the ‘bad cholesterols’, triglycerides and total cholesterol, were observed with adjustment for the basic set of covariates. This is consistent with a number of other studies [7, 33, 36]. Contrary to what may be expected, sedentary behavior time or PA time was not associated with any lipid level in overweight or obese participants. To our knowledge, this has not been demonstrated previously, and suggests that we need to differentiate prevention measures targeting obese subjects.

Time spent engaging in sedentary behaviors has gained considerable interest recently as a risk factor for the metabolic syndrome, CVD, and mortality [14, 15, 37]. Our findings are consistent with a number of studies showing associations between higher sedentary behavior time and poorer cardiometabolic health, as measured by a cluster of risk factors, in Australian [9], US [7], and European adults [18]. Healy and colleagues [9] found objectively-measured sedentary time in an Australian population was associated with a increased metabolic risk score, comprised of a cluster of factors (waist circumference, triglycerides, blood pressure, fasting plasma glucose). The same relations between sedentary behavior time, HDL and triglycerides have also been shown in extensive NHANES data from the US [7].

Our previous research has shown that television viewing time regardless of day of the week, and computer time on a day off, were negatively associated with an overall index of cardiovascular health [18]. From a public health standpoint, this finding is important. A recent large prospective study has provided evidence that different sedentary behaviors may not have the same association with health outcomes [45], and television time, but not sitting at work or during transportation, was associated with overall and cardiovascular mortality. Therefore, reducing sedentary behavior during ‘free or leisure time’ may be a particularly important message to those who have sedentary or computer-based occupations during the working week.

Our findings in normal weight individuals were similar to those of Aadland et al. [33], who examined PA and sedentary behavior with lipoprotein subclass concentrations in a small sample of healthy adults, with the exclusion of obese participants (BMI ≥30 kg/m^2^). In our study amongst normal weight individuals, the strongest associations for sedentary time were with increases in triglycerides, LDL-cholesterol, and total cholesterol. Aadland et al. [33], also showed a strong relationship between moderate to vigorous PA with HDL, which we similarly observed in the whole sample analysis. However, no associations were observed between moderate or intense PA time and lipid levels in the present study when stratified by BMI, compared with the sedentary behavior time variables. Like Aadland et al. [33], our findings suggest that the patterns of associations between the lipoproteins with PA and sedentary behavior time may differ, and this was further highlighted when we stratified by BMI. HDL-cholesterol was the only lipoprotein to relate to both *intense PA* time (positively) and *screen time* (inversely). These relations were however no longer observed when the sample was stratified by weight status.

The present study sample could be considered active, with 70.4 % meeting the American Heart Association recommendations of at least 150 minutes per week of moderate intensity PA [46]. We found fewer relations between the PA time variables and lipids, compared with the sedentary time variables. Those who reported engaging in at least 1 hour per day of *intense PA* had the lowest levels of triglycerides, while the highest HDL-cholesterol levels were observed in those who undertook between 0.5 and 1 hour of intense activity per day. No significant relations were observed with *moderate PA* time. This finding is however, consistent with others [9].

This study presents some novel findings and has several strong points. We utilized recent data from a nationwide, population-based sample, with extensive data on cardiovascular risk factors and other potential confounding variables. We have differentiated between type of sedentary behavior (*screen time* in addition to *overall sitting time*), intensity of PA (*moderate* versus *intense*), and between the occasion (*workday* versus *day off*). Furthermore, we have examined these relations in two groups according to body weight status.

The use of a self-reported measure of sedentary behavior and PA time as opposed to objective, accelerometer-derived measures must be acknowledged. We used the IPAQ, which may underestimate the strength of some relationships with disease risk factors [36] and is subject to reponse bias [47]. However, self-reported measures remain the most feasible and affordable instrument for the global surveillance of PA. As the IPAQ refers to the seven days preceding the interview, it also may be less accurate in reflecting long-term or seasonal behavior patterns. The cross-sectional design precludes any conclusion regarding causality between PA, sedentary behaviors and lipid concentrations. Although we controlled for a number of demographic and socioeconomic variables, diet, alcohol intake and smoking, confounding by other factors could explain the results. Gender was adjusted for; there was no evidence of effects-modification according to gender.

Future studies should explore associations between different sedentary behaviors and varying levels of PA intensity with cardiometabolic risk factors, particularly utilizing objective measures of sedentary time and energy expenditure [48]. The present results, as well as previous study findings [18] indicate that targeting a reduction in screen time on days off, such as time spent watching television and on a computer, may be just as important as interventions aimed at reducing sitting time during the working week.

## Conclusions

\The findings from this cross-sectional study indicate that greater *screen time* may be associated with a poorer lipid profile (notably among normal weight subjects). Reducing sedentary behavior time and engaging in daily intense PA may be of benefit to levels of HDL-cholesterol and triglycerides, and important for the prevention of cardiovascular health problems.
